# Supplementary material for: Content-rich biological network constructed by mining PubMed abstracts
Source: BMC Bioinformatics. 2004 Oct 8;5:147. doi: 10.1186/1471-2105-5-147 (PMC528731; doi:10.1186/1471-2105-5-147)
Supplement: Additional File 5 — The original Chilibot query results of the term "long-term potentiation (LTP)" and 22 other terms, limiting the latest references analyzed to the years 1990, 1995, 2000, and 2004. [file 1471-2105-5-147-S5.bz2 › chilibotAdditionalFile5/ltp2004/html/PI-3K.html]

 


**PI-3K** (Input: PI-3K ) 

---


**Google Searches:** Entire Web  | EDU domain only  | PDF files only

.

**External Links:** OMIM |
LocusLink
| Swissprot
| GeneCards

  
**Maps of PI-3K**

Simple
Complete
graph in
radiant
tree
square
layout.

**New Hypothesis !**

**Synonyms** 

- pi-3k   [PubMed]

- pi-3-kinase   [PubMed]

- pi3-kinase   [PubMed]

**Synopsis**

- Our results suggest that the Src kinase Lyn is involved in PLC gamma 2 phosphorylation and PI 3 kinase  [**PI-3K**]  activation induced by Epo.  Biochem Biophys Res Commun, 2003 
  [23]

- These results suggest that in addition to the positive effects mediated by PI3 kinase  [**PI-3K**]  that are apparent in Gab2 mast cells, Gab2 by interacting with Lyn and PLC gamma may have negative regulatory effects on Fc epsilon RI induced mast cell signaling and functions.  J Immunol, 2002 
  [20]

- These results suggest that both diacylglycerol sensitive PKC, activated by PLC products, and diacylglycerol insensitive PKC, possibly activated by a G i PI 3 kinase  [**PI-3K**]  dependent process, are involved in ET 1 induced ERK activation.  Am J Physiol Cell Physiol, 2002 
  [20]

- In summary, these data indicate that PLCgamma, activated at least partially by PI3 kinase  [**PI-3K**] , is a link between insulin receptor and PKCzeta through the production of PA and could mediate insulin induced glucose uptake and GLUT4 translocation.  Exp Cell Res, 2002 
  [20]

- Moreover, PLC was found to act upstream of the PI 3 kinase  [**PI-3K**]  IA isoform.  Biochem Biophys Res Commun, 2001 
  [18]

- Together, our results indicate that the role of PI3 kinase  [**PI-3K**]  in LTP is NOT limited to its role as an upstream regulator of MAPK signaling but includes signaling through ERK independent pathways that regulate LTP induction.  J Neurosci, 2003 
  [17]

- The evidence presented is consistent with the hypothesis that PI 3 kinase  [**PI-3K**]  activation, possibly by NGF, plays a role in expression of LTP in dentate gyrus.  Neuropharmacology, 2000 
  [16]

- The present study suggests that the PLC signaling pathway, which may be activated by PLC beta and PLC gamma, respectively, and the PI 3K  [**PI-3K**]  signaling pathway are involved in the AA stimulated respiratory burst in human neutrophil.  J Leukoc Biol, 2003 
  [16]

- The autonomous intranuclear inositide cycle is endowed, besides the PLC, with conventional lipid kinases and phosphatidylinositol 3 kinase PI 3 kinase  [**PI-3K**]  which plays an important role in granulocytic differentiation.  Ital J Anat Embryol, 2001 
  [16]

- This suggests that PI 3 kinase  [**PI-3K**]  contributes to the maintenance of synaptic transmission during periods of high activity, indicating a possible role in synaptic plasticity.  J Biol Chem, 2003 
  [16]

- Use of selective inhibitors of signal transduction and the quantitation of the levels of phosphorylated MAPK ERK activating kinase 1 MEK1, extracellular signal regulated kinase 1 ERK1, and p38 mitogen activated protein kinase MAPK suggests that the effects of Cd 2 are mediated by the p21 ras dependent MAPK,  Cell Signal, 2002 
  [16]

- It has been reported that the activation of the extracellular signal regulated kinase 1 2 Erk 1 2 and phosphoinositide 3 PI3 kinase  [**PI-3K**]  plays a crucial role in the NGF induced differentiation of PC12 cells.  Biochem Biophys Res Commun, 2004 
  [16]

- Using immunoprecipitation Western immunoblotting however, we were unable to show any direct association between CD4 and PLC gamma, PI 3K  [**PI-3K**] , or other known signaling proteins.  Exp Cell Res, 2002 
  [15]

- NECA mediated IL 6 release was inhibited by the PLC inhibitor 1 6 17beta 3 methoxyestra,5 10 tiene 17 yl amino hexyl 1H pyrrole dione, the PI3 kinase  [**PI-3K**]  inhibitor wortmannin and the PKC inhibitors bisindolylmaleimide 1 and bisindolymaleimide X1 HCl Ro 32 0432 .  Br J Pharmacol, 2003 
  [15]

- The pathway through which EGFR activates PKCd is suggested by the fact that the MEK 1 inhibitor U0126 and the PI3 kinase  [**PI-3K**]  inhibitor LY294002 had no effect on PKCd activation.  J Biol ChemJ Biol Chem, 2003 
  [13]
